# *Prevotella* as the main driver for the association between dairy farming and human gut microbiome composition

**DOI:** 10.3389/frmbi.2025.1612922

**Published:** 2025-07-09

**Authors:** Tryntsje Cuperus, Jolanda Kool, David Boverhoff, Kees van der Ark, Marieke Opsteegh, Susana Fuentes

**Affiliations:** Centre for Infectious Disease Control, National Institute for Public Health and the Environment (RIVM), Bilthoven, Netherlands

**Keywords:** gut microbiome, cattle, livestock, dairy farming, *Prevotella*, occupational exposure, farmers, One Health

## Abstract

The human gut microbiota is shaped by a multitude of environmental factors, including contact with animals. To investigate the association between occupational exposure to cattle and the gut microbiome, a cross-sectional study was performed on 65 individuals working and/or living on Dutch dairy cattle farms in comparison to controls. The gut microbiome of the participants was assessed by 16S rRNA gene amplicon sequencing of stool samples. A lower alpha diversity and divergent microbiome composition was observed, driven largely by a greater *Prevotella* abundance in dairy farm participants when compared to controls. *Prevotella* was also associated with contact frequency with the dairy cattle, with participants with more frequent contact showing higher abundance. The results of this study show occupational contact with cattle is associated with gut microbiome composition, which is of relevance because of the importance of the microbiome for human health.

## 1 Introduction

The bacterial communities in the gastro-intestinal tract of animals and humans have a multitude of functions that can impact health, such as the metabolism of nutrients and interactions with the immune system. The composition of the gut microbiota is shaped by both intrinsic and extrinsic or environmental factors (e.g., diet and geography) (Spor et al., 2011; Gupta et al., 2017). The human-animal interface is an important element of our environment, and interactions between humans and animals are well-recognized as important determinants for public health. Especially when this relates to zoonoses, as up to 60% of human pathogens are of zoonotic origin (Rahman et al., 2020). Exposure to animals also includes exposure to their microbiomes. In recent years, studies have shown that contact with animals can have an influence on the microbiome of humans (Trinh et al., 2018; Mucci et al., 2022). Among others, influence on the diversity and composition of skin, nasal and gut microbiota has been described for people living together with pets or having occupational contact with livestock. Also, microbiome and resistome (i.e., all the antibiotic resistance genes in the gut ecosystem) components can be shared between humans and animals when in close contact (Sun et al., 2020; Yang et al., 2023; Mahmud et al., 2024). In the Netherlands, dairy farming is an important livestock sector with more than 15–000 dairy farms and 1.5 million dairy cattle in 2021 (CBS, 2025). Often, these are family owned farms, where contact of the farmer and family members with the dairy cattle is frequent. In previous studies from other countries, influence of contact with dairy cattle on the nasal and gut microbiome of humans has been described (Shukla et al., 2017; Mahmud et al., 2024). In this pilot study we aimed to compare the gut microbiome of participants from Dutch dairy farms with age and sex-matched control subjects from a population-wide study to determine the main drivers of the microbiome composition. This will help to elucidate the impact of occupational animal exposure on the human gut microbiome.

## 2 Material and methods

### 2.1 Study design

The samples used in this study are part of a surveillance program for zoonotic pathogens in livestock (Cuperus et al., 2022). In short, 200 dairy farms with a minimal farm size of 50 adult dairy cattle, were selected for farm visits using probability sampling without replacement (i.e. probability of inclusion increased with farm size). Dairy farms were visited and cattle samples (faecal samples and skin swabs) were taken to analyse for multiple zoonotic pathogens (van Duijkeren et al., 2025; Cuperus et al., 2024). In addition to the animal samples, dairy farmers, their family members and employees, aged 18 or up, were asked to participate in the human study. Multiple participants from each farm were allowed to participate. Informed consent was obtained from the participants. According to the Dutch Medical Research Involving Human Subjects Act (WMO) this study was exempt from review by an Institutional Review Board.

### 2.2 Sample collection

Participants were sent a study kit between February and September 2021, with materials to collect a faecal sample in a DNA/RNA Shield Fecal Collection Tube (Zymo Research). The study kit also contained instructions on proper sample collection. Participants were asked to return the faecal sample, in a safety bag and medical envelope (UN3373 compliant), by regular mail to the Dutch Institute for Public Health and the Environment. Samples were received within three days from collection and frozen at -80°C upon arrival. Control samples were collected from healthy participants in a similar way.

Informed consent was obtained from the participants. Participants who reported to have used antibiotics within six months before sampling or that suffered from chronic intestinal complaints were excluded. Control participants (i.e., not dairy farmers), were selected from two Dutch population-wide studies on vaccine efficiency, before the vaccination event (RIVM 2024a, 2024b). Control subjects were matched to the dairy farmers based on sex and age. For 36 dairy farm participants a matched control was found where the age deviated five years at maximum. For the remaining dairy farm participants the control was found in the same age group (18–59 year or ≥60 years). The average age of the DF participants was 49 (range: 18–75 years) and from the controls 47 (range: 14–77 years). The male/female ratio was 65/35% in both groups.

### 2.3 DNA isolation

The Maxwell RSC Blood DNA extraction kit was used according to manufacturer’s instructions with several modifications. One ml of well-homogenized faecal material was added to 0.1 mm zirconia/silica beads and 2.5 mm glass beads. The faecal suspension was mechanically disrupted three times for one minute in a FastPrep-24 Instrument at room temperature and 5.5 oscillations, and maintained on ice after every cycle. Samples were further heated at 95°C for 15 minutes shaking at 300 rpm, and centrifuged for 5 minutes at full speed. Resulting supernatants (faecal lysates) were collected and the pellet was further resuspended in an additional 350 µl of S.T.A.R. buffer following the same procedure. Pooled faecal lysates were then transferred to the Maxwell RSC Instrument (Promega Benelux BV) for further purification steps. Eluted sample was cleaned-up using the OneStep PCR Inhibitor Removal Kit (Zymo Research), total DNA was measured using a Quantus fluorometer (Promega), and the bacterial load was quantified using a quantitative PCR using universal 16S rRNA primers (Eub341F and Eub534R (Muyzer et al., 1993). Every extraction round included two negative DNA extraction controls (blank samples with S.T.A.R. buffer without any added faecal material) and two microbial mock communities as positive controls (ZymoBiomics Microbial Community Standards; Zymo Research).

### 2.4 Illumina sequencing

The concentrations of bacterial yield obtained from the quantitative PCR were used to equalize and dilute the amount of bacteria in all samples to an input of 100 pg DNA. The V4 region of the 16S rRNA gene was amplified, using the 515F (5’- GTG CCA GCM GCC GCG GTA A-3’) and 806R (5’-GGA CTA CHV GGG TWT CTA AT-3’) primers, including the Illumina flow cell adapter and a unique 8-nt index key (Kozich et al., 2013; Caporaso et al., 2011). Additional negative controls (MilliQ) and microbial mock community samples were added during PCR and sequenced alongside the samples. Fragments of the amplified product were quantified using the QIAxcel DNA High Resolution Kit on the Qiaxcel Advanced System (Qiagen) and pooled equimolar. The pool was purified twice, using AMPure XP magnetic beads (Beckman Coulter). KAPA library quantification kit (Roche) was used for the final quantification of the pool to determine the exact input for the Paired-end sequencing, using a V3 Miseq reagent kit (600 cycles) on a Illumina Miseq instrument (Illumina).

### 2.5 Data analysis

Raw sequencing data were quality checked and taxonomically classified using the DADA2 pipeline (Callahan et al., 2016) using default parameters. Analysis of sequencing data was performed in R version 4.1.0. Alpha and beta diversity were calculated using the phyloseq package in R (McMurdie and Holmes, 2013). Significance for differences in alpha diversity was calculated using Wilcoxon test within the *stat_compare_means* function in the ggpubr R package (Kassambra, 2023). PERMANOVA, calculated using the *adonis* function in the vegan R package (Oksanen, 2022), was used for differences in beta diversity. For the plotPCoA analysis, Bray-Curtis dissimilarity was calculated using genus-level data and visualized with the plotPCoA function from the biomeViz package (Shetty, 2025). Genera associated with the PCoA axes were identified using Spearman correlation between their abundances and sample coordinates.

## 3 Results

In this study, the faecal microbiome from 130 persons was determined; 65 participants from dairy farms (DF) and 65 controls. DF participants originated from 36 different farms (1–4 participants per farm). From the DF participants, 63% reported being a dairy farmer with the others being relatives (partner, parent or child), and one person reported being an employee. Most of the DF participants (92%) reported going into the cattle stables ≥ 1 time a day, and 82% reported daily physical contact with cattle. For the controls, no information on animal contact was available.

**Figure 1:**
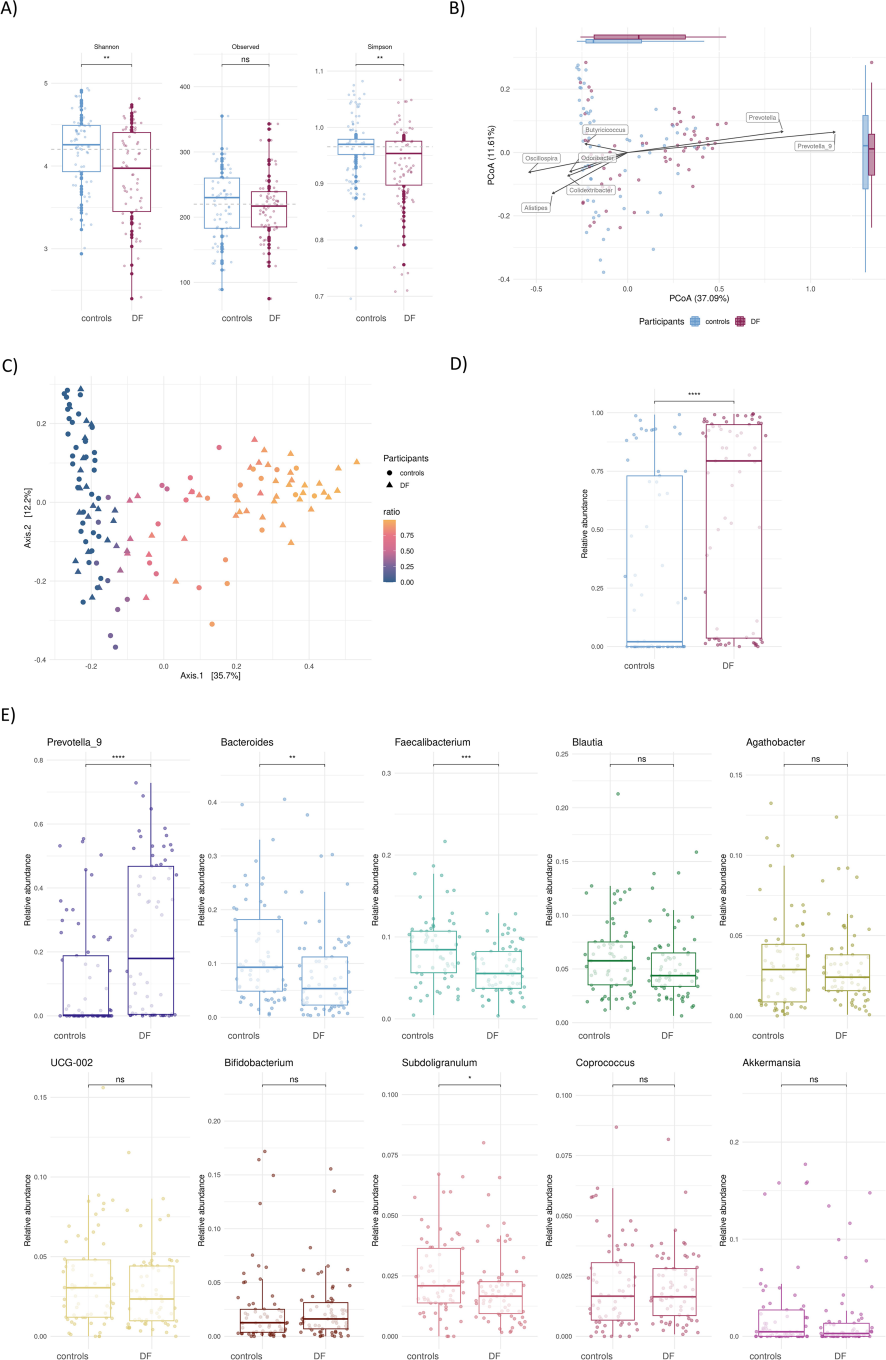
Gut microbiome composition of dairy farm participants (DF) and controls. **(A)** Alpha diversity shown as Shannon, Observed and Simpson indices, **(B)** Principal Coordinate Analysis (PCoA) comparing microbiome composition of DF participants and controls. The side and top boxplots summarize the distribution of sample coordinates along the first two PCoA axes, showing how each group varies in these dimensions. Arrows represent genera that significantly correlate with the PCoA axes, identified using Spearman correlation between their abundances and sample coordinates, indicating their contribution to sample separation, **(C)** PCoA of *Prevotella*/*Bacteroides* ratio of all participants, **(D)** Boxplot comparing *Prevotella/Bacteroides* ratio of DF participants and controls, **(E)** Comparison of the relative abundance of the top 10 genera between the study groups. ns=not significant, p>0.05, *=p<0.05, **=p<0.01, ***=p<0.001, ****=p<0.0001.

Alpha diversity of DF was lower compared to controls, both in the Shannon (p=0.0059) and Simpson index (p=0.0018), with no differences in the observed number of taxa (p=0.45, **Figure 1A**). The overall microbiome composition of the DF participants was significantly different from the controls, assessed by the Bray Curtis dissimilarity index and Principal Coordinate Analysis (PCoA) (PERMANOVA p=0.001, **Figure 1B**). Beta diversity analyses showed that the difference between DF participants and controls was driven largely (with 37% of variance explained by the first principal component) by the higher relative abundance of *Prevotella* and *Prevotella_9* groups (p=2.8x10^-6^, with amplicon sequence variants (ASV’s) further annotated as *P. copri*) (**Figures 1B, E**).

Looking at the ratio of *Prevotella* to *Bacteroides* in our study population, a clear gradient emerged, which was distinctive between DF participants and controls (**Figures 1C, D**). At the genus level, the other significant differences were a higher relative abundance of *Bacteroides* (p=0.0043), *Faecalibacterium* (p=0.0004) and *Subdoligranulum* in the controls (p=0.047, **Figure 1E**).

**Figure 2:**
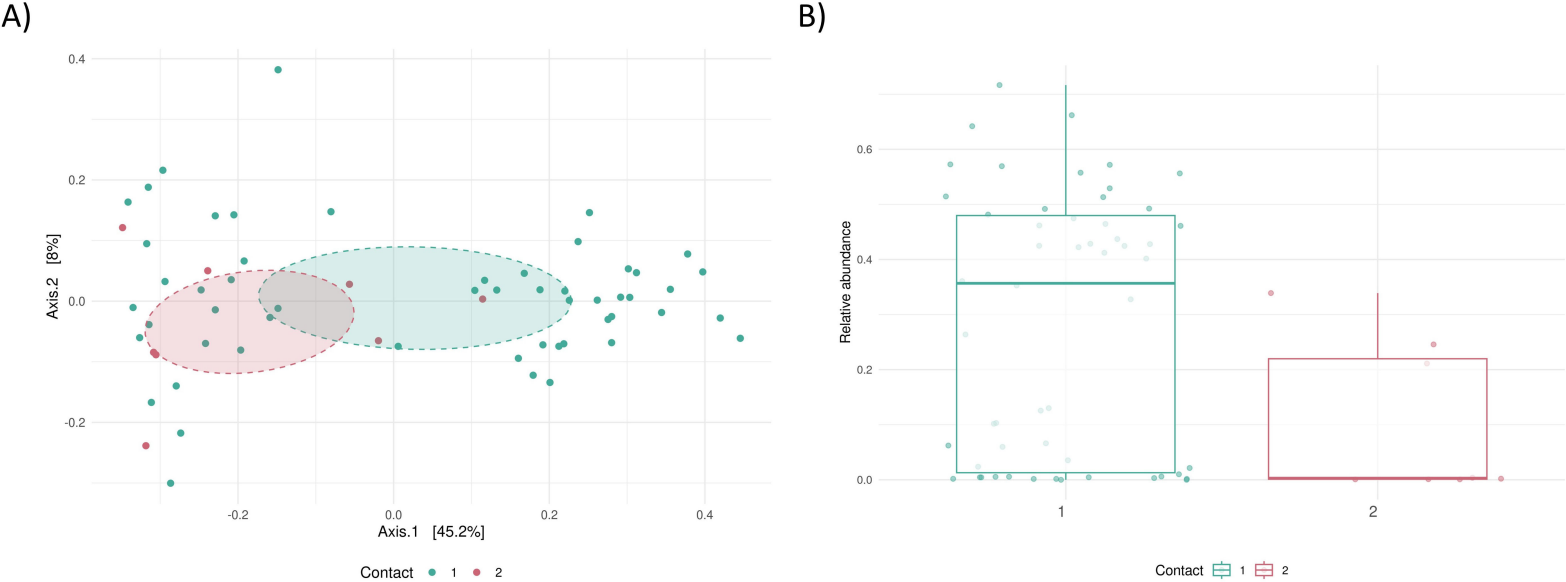
Association of contact frequency with dairy cattle and microbiome composition. **(A)** PCoA by contact with cattle 1: once a day or more, n=57 vs. 2: once a week or less, n=8, **(B)** Relative abundance of *Prevotella*_9 in the groups.

We further investigated the influence of contact frequency (i.e., contact with cattle once a day or more, n=57 vs. once a week or less, n=8). Although group size was limited, we observed significant differences in both overall microbiome composition (PERMANOVA p=0.019, **Figure 2A**) and in the relative abundance of *Prevotella*_9 between the groups (p=0.01, **Figure 2B**). In the group with less contact with cattle, the relative abundance of *Prevotella_9* was significantly lower.

## 4 Discussion

In this study, we compared faecal microbiome composition between participants from Dutch dairy farms and control subjects. The alpha diversity of DF participants was lower than controls. Previous reports on farming and alpha diversity have been conflicting, as a study in Chinese pig farmers showed a higher diversity in control subjects, while a Dutch study on pig farmers and a US study on dairy farmers did not report differences compared to the control subjects (Sun et al., 2017; Van Gompel et al., 2020; Mahmud et al., 2024). While a lower microbial diversity has been associated with diseased states, in these cases, other than in our study, a lower diversity was observed together with an increased proportion of facultative anaerobes (e.g. *Proteobacteria* and *Bacilli*), often considered less desirable (Kriss et al., 2018).

Overall microbiome composition was significantly different between DF participants and controls. In contrast, in the study among US dairy farmers, no difference in overall microbiome composition was observed compared to controls (Mahmud et al., 2024).

Principal coordinate analysis showed that the main driver of the difference between DF and control microbiomes was the genus *Prevotella*. Members of the genus *Prevotella* are Gram-negative anaerobic bacteria, found in various animal hosts. In ruminants, *Prevotella* are a common feature of the gut microbiome (Henderson et al., 2015). Human gut microbiomes are often dominated by either *Prevotella* or *Bacteroides* (Gorvitovskaia et al., 2016), with *Prevotella*-dominated microbiomes more often found among non-Western populations. In addition, a high abundance of *Prevotella*, a fibre-degrading genus, is also associated with diets rich in fibres and complex carbohydrates (Tett et al., 2021). A limitation of our study was the lack of diet and other lifestyle information gathered from both DF participants and controls. The potential contribution of these factors, especially diet, to the *Prevotella* abundance remains to be determined. Potential associations of *Prevotella* with health and disease are currently still unclear, with conflicting reports linking this genus to both beneficial or detrimental health outcomes (Abdelsalam et al., 2023). It becomes more evident that there is also a link between *Prevotella* and animal contact, as higher *Prevotella* abundance was previously observed in a US study of dairy farmers, a Swiss study of pig farmers and associated with pet ownership in a Dutch cohort (Moor et al., 2021; Mahmud et al., 2024; Gacesa et al., 2022).

Differences in microbiome composition and *Prevotella* abundance were not only observed between DF participants and controls, but also between DF participants with frequent and less frequent contact with the dairy cattle. These findings suggest that the observed associations between dairy farming and gut microbiome are most likely a direct effect of (frequent) contact with dairy cattle, rather than other possible differences between DF and controls. In a previous study in pig farmers, common bacterial sequences, including *Prevotella*, were found in samples from the farmers, the pigs and air samples from the stables (Moor et al., 2021). The authors suggested that farmers took up aerosols with bacteria derived from the pigs. Other studies also reported overlap of sequences between humans and the animals they were in close contact with (Tan et al., 2020; Mahmud et al., 2024). Possibly, shared microbial lineages between the dairy cattle and DF participants, leading to a shift in microbiome composition, could be similarly explanatory for our findings. Unfortunately, we did not include samples from the dairy cattle in our study, as these samples were not suitably collected for microbiome analysis (e.g., to preserve nucleid acids or prevent the overgrowth of anaerobes). This could be a valuable addition for future studies as insight in shared sequences at farm-level would help further explain the microbiome diversity in dairy farmers.

In conclusion, we report an association between dairy farming and the gut microbiome of farmers and their family members, largely driven by *Prevotella*, and likely as a direct effect of contact with dairy cattle. Our results are an addition to previous studies and strengthen the knowledge about the influence of human-animal contact on the microbiome.

## Data Availability

The datasets presented in this study can be found in online repositories. The names of the repository/repositories and accession number(s) can be found below: https://www.ebi.ac.uk/ena, PRJEB88067.
